# Should neurologists initiate treatment for hypertension and hyperlipidemia to reduce cardiovascular risk in epilepsy?

**DOI:** 10.3389/fneur.2023.1268920

**Published:** 2024-07-04

**Authors:** Mark Gaertner, Christopher M. DeGiorgio

**Affiliations:** ^1^Department of Neurology, David Geffen UCLA School of Medicine, Los Angeles, CA, United States; ^2^Olive View-UCLA Medical Center Sylmar, Los Angeles, CA, United States

**Keywords:** epilepsy, hypertension, hyperlipidemia, cardiovascular risk, ACC-ASCVD risk score, neurologist

## Cardiovascular related mortality in epilepsy

Cardiovascular disease and stroke are major causes of death in epilepsy (1, 2). People with epilepsy are at higher risk for developing ischemic heart disease and stroke (3). Epilepsy is independently associated with a higher incidence of myocardial infarction when adjusted for age, gender, and risk factors (4). There are likely several causes for the increased cardiovascular risk in epilepsy, including an increased prevalence of diabetes, hypertension, obesity, and hyperlipidemia (5). Terman et al. performed a retrospective study of 17,961 persons ages 18 or higher enrolled in US National Health and Nutrition Examination Survey (NHANES survey) for the years 2013 and 2018 (5). One hundred and fifty-four individuals reported a history of epilepsy, defined as exposure to at least one antiseizure medication in the last 5 years (5). Those with epilepsy had significantly higher rates of hypertension, diabetes, heart disease and stroke than those without epilepsy (5). Ten-year Atherosclerosis Cardiovascular Risk Score (ASCVD score) was significantly higher in people with epilepsy then those without epilepsy; the mean 10-year ASCVD risk score was 6.1% (SD 8.2%) in epilepsy, vs. 5.2% (SD 8.3%, *p* < 0.05) in those without epilepsy (5).

Additionally, there is recent research suggesting that there may be a pivotal role of certain anti-hypertensive medications in potentially preventing epilepsy in animal models. Dong et al. showed that the angiotensin converting enzyme (ACE) inhibitor captopril prevented the development of kainic acid (KA) induced status epilepticus in rats when co-administered (6). The rats were also noted to have improved cognitive outcomes compared to the KA only treated group. The proposed mechanism involves a reduction in microglia-dependent synaptic remodeling dependent on complement C3a which was partially blocked by administration of recombinant C3a supporting a potential anti-inflammatory role of ACE inhibition in preventing the development of seizures (6).

## Factors that may contribute to healthcare outcome disparities in epilepsy

Socioeconomic, racial, and ethnic disparities for cardiovascular risk factors may be contributors (7–9). People with epilepsy are more likely to be enrolled in Medicaid or underinsured, and more likely to experience delays to epilepsy diagnosis and treatment (7, 8). People with epilepsy report more difficulty affording medications and may reduce or skip medications to save money (9). People with uncontrolled epilepsy are more likely to access care via emergency rooms (8–10). In patients with acute seizures presenting to the emergency room, we can hypothesize that cardiovascular risk factors may not be fully addressed since the focus is to stop seizures. When people with epilepsy do see neurologists, cardiovascular risk factors surveillance may be deferred since the neurologist's primary role is to diagnose and manage seizures in a time-constrained environment.

A lack of surveillance and treatment of cardiovascular risk factors by neurologists may result in missed opportunities to prevent heart disease, diabetes, and stroke in epilepsy (11, 12). When Neurologists do treat hypertension, outcomes are improved (12). As stated in a recent editorial by Estol, commenting on the failure of neurologists to treat hypertension*, “[Neurologists] dropped the hammer on hypertension 20 years ago!”* (12). Similar to the data reported by Estol, we identified a cohort of 93 patients with epilepsy ages 40 and older from primary care, the emergency room or hospital. 58.1% had hypertension and 80.6% had hyperlipidemia, of whom 35.2 and 49.4%, respectively were untreated. Fifty percentage had a BMI > 30% (obese), and the mean HbA1C level was 6.0% (prediabetic, normal < 5.7%). This data set indicates there is an opportunity for neurologists to identify at risk patients and either alert primary care physicians or initiate treatment in a collaborative fashion. Given the evidence that early treatment of mild HTN reduces all-cause mortality, stroke, and cardiovascular disease (12), a more active role in by neurologists may bridge gaps and reduce the burden of cardiovascular disease in people with epilepsy.

## A potentially pivotal role for neurologists in reducing cardiovascular risk

To accomplish this goal, a new paradigm needs to be explored. In this new model of care, neurologists take an active role in monitoring or modifying key variables such as blood pressure and lipids (13). Neurologists can use simple algorithms provided by the American College of Cardiology (14, 15) and access guidelines published by the American Heart Association, which are readily available as downloadable Apps for IOS and android operating systems. One tool that neurologists can access is the American College of Cardiology 10-year ASCVD tool (14, 15), linked here. ASCVD Risk Estimator + (acc.org). Or rather, neurologists can alert primary care to untreated risk factors and act as accelerants to reduce cardiovascular risk. This may be especially important in underserved population, where gaps in care are prevalent.

This new paradigm will require an evidence-based approach as well as increased awareness and education from major organizations such as the American Academy of Neurology, the American Epilepsy Society, the Epilepsy Foundation and epilepsy advocates worldwide. Funded research studies should be conducted comparing the outcomes of neurologist-initiated treatment vs. routine care. Actions may include creating special interest groups and education sessions to help neurologists become more familiar with cardiovascular risk factor reductions; indications, side effects and pharmacology of anti-hypertensive and lipid lowering agents (13); and easy-to-use online tools. We believe the time has come for neurologists to get more educated and engaged in cardiovascular risk reduction to benefit our patients. The need is great and the tools are here to make this a reality.

## Author contributions

MG: Writing—review and editing. CD: Conceptualization, Writing—original draft.
